# Coexistence of primary pulmonary meningioma and metastatic papillary renal cell carcinoma of the lung: A rare case report with review of the literature

**DOI:** 10.1097/MD.0000000000032157

**Published:** 2022-12-16

**Authors:** Hao Tang, Yutao He, Long Wang, Guomin Wu, Lina Wang, Yujuan Xu, Deyu Guo

**Affiliations:** a Department of Pathology, Guiqian International General Hospital, Guiyang, Guizhou Province, China; b Department of Laboratory Medicine, Guiqian International General Hospital, Guiyang, Guizhou Province, China.

**Keywords:** clinicopathological features, lobectomy, papillary renal cell carcinoma, primary pulmonary meningioma

## Abstract

Primary pulmonary meningioma (PPM) is extremely rare tumor and only a few reports have been reported to date. PPM may be overlooked when it coexists with other types of tumors in the lung. It is essential to have a knowledge of the clinicopathological features of PPM and to recognize this rare coexistence.

**Patient concerns:**

A 57-year-old male underwent surgery for papillary renal cell carcinoma, when 2 pulmonary nodules were detected using chest computed tomography.

**Diagnosis:**

The coexistence of benign PPM and metastatic papillary renal cell carcinoma was histologically confirmed.

**Interventions:**

A lobectomy was performed.

**Outcomes:**

The patient recovered well after surgery and was discharged on postoperative day 4.

**Lessons:**

Duo to the rarity of PPM, it is easily overlooked, especially when it coexists with other tumors in the lung. The possibility of PPM needs to be taken into account when diagnosing pulmonary nodules in clinical practice.

## 1. Introduction

Meningiomas are an extraordinarily common and usually benign primary brain neoplasms, with the proportion of approximately 37.6% of all primary neoplasms of central nervous system (CNS).^[1]^ Primary ectopic meningiomas, accounting for 1% to 2% of all meningiomas, typically occur in the head and neck sites, including nose, paranasal sinus, orbit, oropharynx and cranium.^[2]^ Primary pulmonary meningioma (PPM) is even more uncommon and usually presents as a single well-circumscribed nodule on imaging.^[3]^ Since the first case of PPM was delineated in 1982, merely 68 cases had been described in the medical literature.^[4]^ Furthermore, with the exception of merely 2 cases with contralateral lung adenocarcinoma, PPM usually develops independently in the lung.^[3,5]^

Papillary renal cell carcinoma (pRCC) is the second most common subtype of renal cell carcinoma (RCC), accounting for 10% to 15% of all cases.^[6]^ Similar to clear cell RCC, pRCC most often metastasizes to the brain, bone, lung and liver.^[7]^

In consideration of the extreme rarity of PPM, it is most likely to be overlooked in clinical practice especially when they coexist with other lung neoplasms. Herein, we described a case of a PPM accompanying metastatic pRCC as a reminder of the possibility of this extremely rare coexistence to avoid misdiagnosis, and reviewed relevant literature for the purpose of further understanding this extraordinarily unusual tumor of PPM.

## 2. Case report

**Figure 1.**
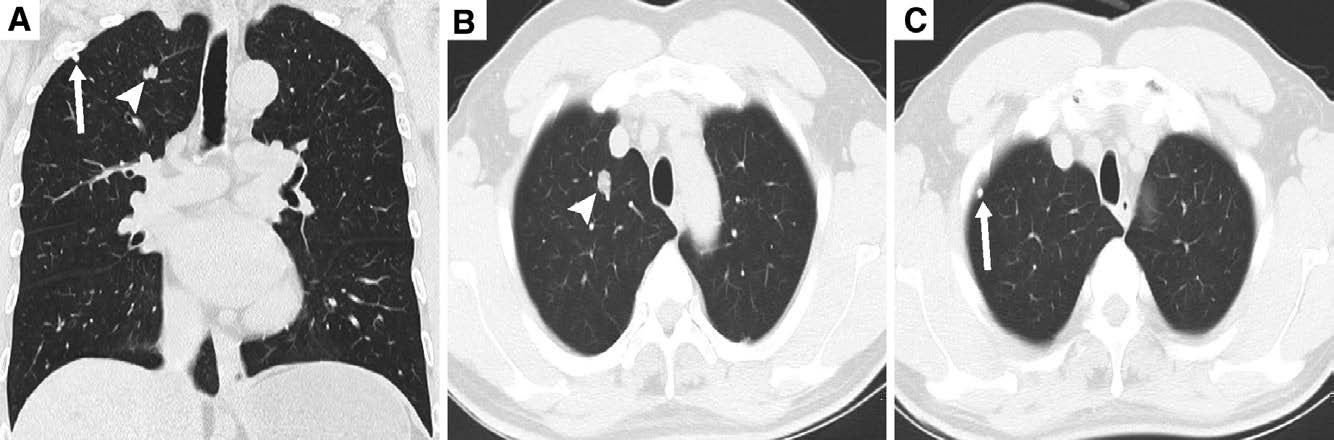
(A) Thoracic CT scan described 2 well-circumscribed pulmonary nodules in the right upper lobe. (B, C) These 2 lesions were located at the apex of the lung (white arrow-head) and on the surface of visceral pleura (white arrow) respectively.

A 57-year-old asymptomatic male was diagnosed with type 1 pRCC by surgically resected specimen whereas 2 well-circumscribed solitary solid pulmonary nodules were discovered in the upper lobe of the right lung by thoracic computed tomography (CT) (Fig. 1A). One nodule located at the apex of the lung, measuring 15 mm × 9 mm without calcification and pleural invasion (Fig. 1B). Another 5 mm diameter nodule located on the surface of visceral pleura with diffuse calcification (Fig. 1C). After 10 months of follow-up, the size of these 2 lung nodules did not change significantly on radiological studies. Subsequently, a right upper lobectomy was performed in order to understand the nature of these 2 lesions.

**Figure 2.**
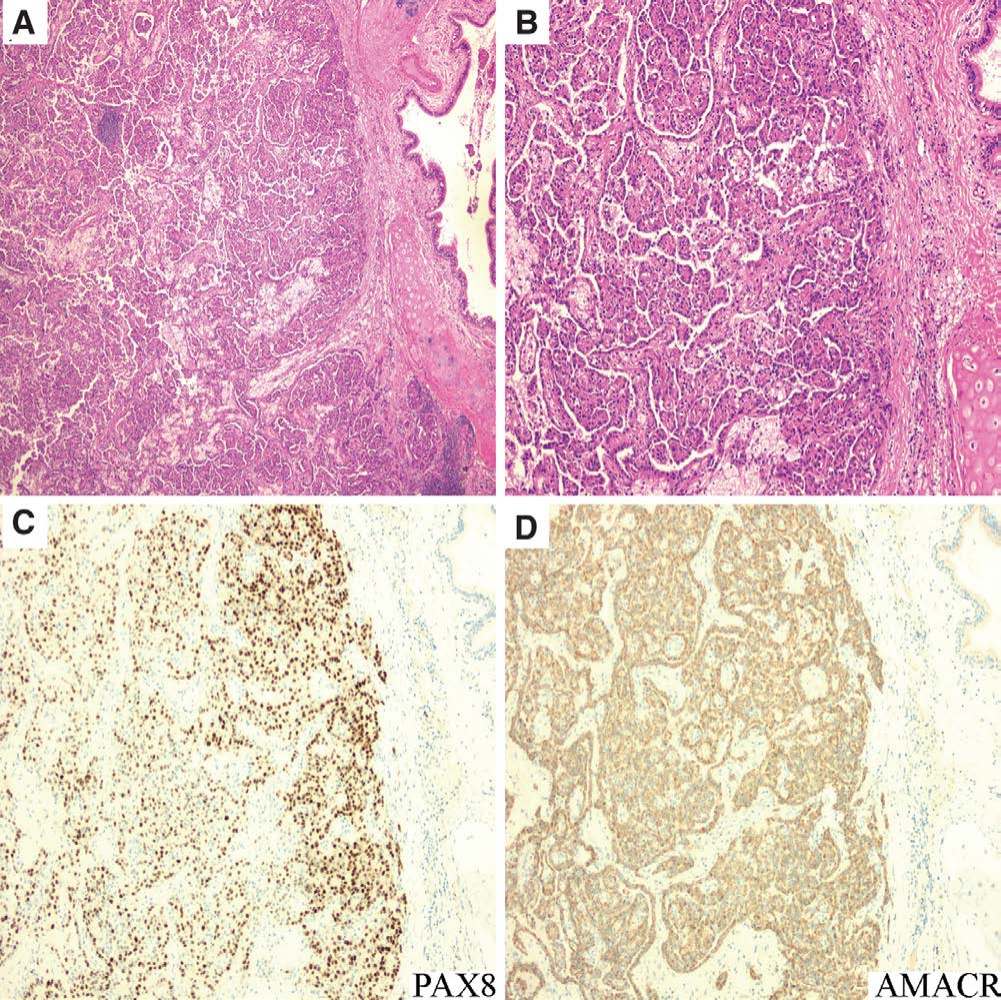
HE and immunohistochemical staining findings of the nodular lesion at the apex of the right lung. (A) This nodular lesion showed typical pathomorphology of classically type 1 papillary renal cell carcinoma (pRCC) with papillary or tubulopapillary growth pattern and aggregates of foamy macrophages in the axis of papillae (Hematoxylin and Eosin stain (H&E) 100 × magnification). (B) The neoplasm was composed of columnar or cuboidal epithelial cells and arranged in a shallow layer, with minimal cytoplasm, small ovoid nuclei, and delicate nucleoli (H&E 200 × magnification). (C, D) Immunohistochemical staining was extensively positive for PAX8 and AMACR (100 × magnification).

Macroscopically, the nodular lesion at the apex of the right lung, measuring 15 mm × 10 mm × 5 mm, showed gray, solid, well-demarcated and medium texture. Additionally, the nodular lesion on the surface of visceral pleura, with a diameter of 5 mm, exhibited gray, solid, well-demarcated and slightly hard texture. Microscopically, the nodular lesion at the apex of the right lung showed papillary or tubulopapillary growth pattern with sharp boundary from the uninvolved lung (Fig. 2A). The papillae were covered by a single-layer columnar or cuboidal epithelial neoplasm cells with scanty pale cytoplasm, small ovoid nuclei, and delicate nucleoli (Fig. 2B). Moreover, aggregates of foamy macrophages were discovered in the axis of papillae (Fig. 2B). Immunohistochemically, the neoplasm cells were diffuse positive for PAX8, AMACR (Fig. 2C, D), AE1/AE3, and Vimentin. In light of these medically historical, histological and immunohistochemical findings, a diagnosis of metastatic type 1 pRCC was established.

**Figure 3.**
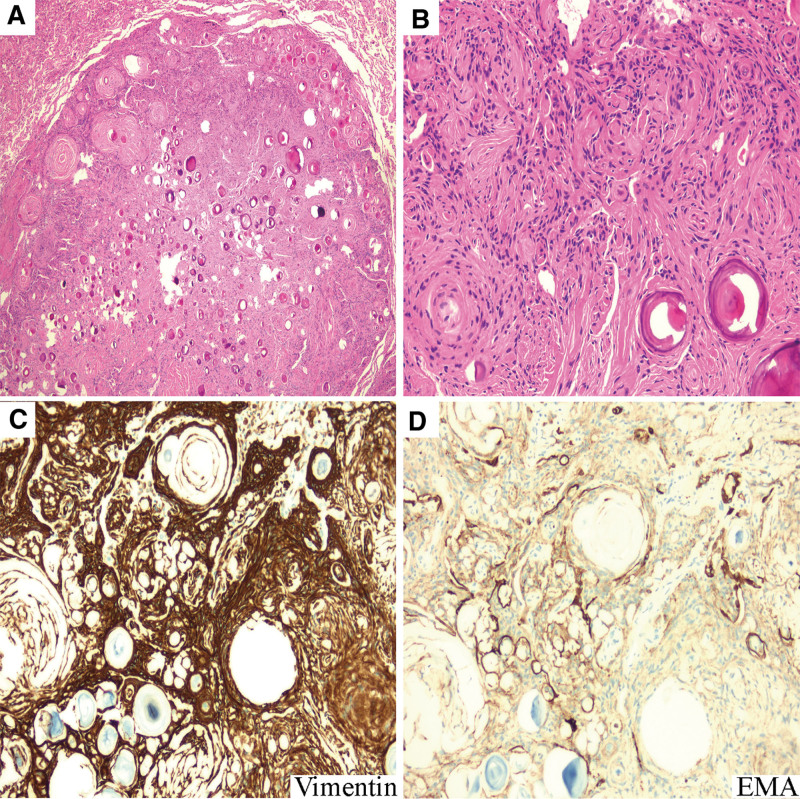
HE and immunohistochemical staining findings of the nodular lesion on the surface of visceral pleural. (A) The sharply demarcated neoplasm was accompanied by the formation of whorl, collagenous nodules and abundant psammoma bodies (H&E 40 × magnification). (B) The tumor cells were fusiform or oval (H&E 200 × magnification). (C, D) The immunohistochemistry examination delineated diffuse strong positive expression of Vimentin and positive expression of EMA (200 × magnification).

The boundary of the nodular lesion on the surface of visceral pleura was clear and the fusiform or oval tumor cells arranged in whirlpool shape with collagenous nodules and abundant psammoma bodies (Fig. 3A, B). The tumor cells were diffuse strong positive immunoreactivity for Vimentin and weakly positive immunoreactivity for EMA (Fig. 3C, D), SSTR2 and PR. Meanwhile, there were no atypical cells and mitotic figures. The patient was assessed with CT of the CNS for possible CNS meningioma, but no abnormalities were observed. According to these radiologic, histological and immunohistochemical results, the pathological diagnosis was given as benign PPM.

In the end, the finally pathological diagnosis of coexistence of benign PPM and metastatic pRCC of the lung was rendered. And the patient recovered well after surgery and was discharged on postoperative day 4.

## 3. Discussion

Meningioma is the most widespread intracranial neoplasm, which rarely metastasizes to the lung, and PPM is more scarce.^[8]^ PPM is more likely to occur in middle-aged and older women.^[9]^ Although most patients are asymptomatic, some may have symptoms such as chest pain, coughing and hemoptysis.^[10]^

In the most cases, PPM is usually found incidentally and often appears as solitary solid pulmonary nodules on chest CT scan.^[11]^ It has been reported that the malignant PPM is not associated with increased positron emission tomography/computed tomography metabolic activity and therefore the use of positron emission tomography/computed tomography is considered to be a suboptimal option for these lesions.^[12]^ These lesions are usually slow-growing benign lesions, and only 7 cases of malignant PPM have been reported.^[13–16]^ Benign PPM typically ranges from 4 mm to 60 mm in diameter.^[17]^ Malignant PPM is usually similar in diameter, with only 1 case measuring 150 mm in diameter.^[16]^ These malignant cases usually show malignant features, including increased mitotic activity, pleural invasion, neoplastic necrosis, lymph node metastasis, or distant metastasis.^[18]^ There are usually no clear predilection for the location of PPM in the lung.^[19]^

Segmentectomy, lobectomy and wedge pneumonectomy are common surgical methods for such lesions.^[8]^ Benign PPM normally has a favorable prognosis after operation, with no recurrence or metastasis. However, malignant PPM may develop distant metastasis or postoperative recurrence.^[20]^

Currently, PPM is thought to have 2 histological origins, such as subpleural multipotential stromal cells and ectopic arachnoid cells.^[5]^ Many pathological differential diagnoses need to be considered for PPM, including sarcomatoid mesothelioma, spindle cell thymoma, spindle cell carcinoma, solitary fibrous tumor, inflammatory myofibroblastic tumor, epithelioid hemangioendothelioma, synovial sarcoma, as well as metastatic tumor.^[5]^

RCC, which comprises 2% to 3% of all cancers, is particularly aggressive and frequently metastasizes to the brain, lung, liver and bone.^[21]^ The lung is the most frequent anatomical site of metastasis for RCC, making up 45% of all metastatic cases.^[22]^ RCC has a multitude of distinct histological types, the commonest of which is clear cell RCC, and this histological type makes up approximately 75% of all primary renal tumors.^[23]^

pRCC is the second most common subtype of RCC, accounting for 10%-15% of all RCC cases.^[24]^ Based on morphological features, pRCC can be subdivided into type 1 and type 2.^[25]^ The histology of type 1 pRCC is characterized by unilaminar arranged small cells with sparse cytoplasm covering the basement membrane of the papillary, and foamy macrophages are commonly observed in its papillary axis.^[25]^ Type 2 neoplasms are typically characterized by relatively large cells with abundant eosinophilic cytoplasm, pseudostratified cell nucleus and large nucleoli.^[25]^ Pulmonary metastases from RCC usually present as solid nodules or masses on CT, which show varying degrees of enhancement.^[26]^

With the exception of barely 2 cases of concomitant contralateral lung adenocarcinoma, PPM is reported to be usually non-coexistent with other tumors in the lung, even in patients with a medical history of malignancy.^[4]^

In summary, despite the infrequency of PPM, the possibility of PPM should be considered in clinical work. We herein presented an extremely scarce case of metastatic pRCC coexisting with a PPM in the lung, which may be a challenge to clinicians in daily clinical practice. It is crucial to be aware of the clinicopathological features of PPM and this uncommon concomitant condition.

## Acknowledgments

We would like to thank the patient and her family.

## Author contributions

**Conceptualization:** Hao Tang.

**Data curation:** Yutao He.

**Formal analysis:** Yutao He.

**Investigation:** Long Wang.

**Methodology:** Long Wang, Guomin Wu, Lina Wang, Yujuan Xu.

**Project administration:** Guomin Wu.

**Writing – original draft:** Hao Tang.

**Writing – review & editing:** Hao Tang, Deyu Guo.
